# The princess at the conference: Science, pacifism, and Habsburg society

**DOI:** 10.1177/0073275320977750

**Published:** 2021-01-14

**Authors:** Geert Somsen

**Affiliations:** History Department/STS Program, Maastricht University, Netherlands

**Keywords:** Bertha von Suttner, scientific internationalism, science fiction, science and pacifism, scientific conferences, late Habsburg culture, aristocratic internationalism

## Abstract

Historians are showing increasing interest in scientific internationalism, the notion that science transcends national differences and hence advances peace and cooperation. This notion became particularly popular in the decades around 1900, the heyday of the universal expositions and the so-called first era of globalization. In this article I argue that in order to properly historicize scientific internationalism, it is imperative to understand how actors *imagined* science to have pacifist effects, and to relate their technoscientific to their geopolitical imaginaries. To illustrate this, I analyze the 1911 novel *Der Menschheit Hochgedanken* (translated as *When Thoughts Will Soar*) by the famous Austrian pacifist Baroness Bertha von Suttner. It tells the story of a scientific conference whose participants, by the sheer brilliance of their thought, ward off war and preserve world peace. Relating the novel to von Suttner’s own life experiences, I situate her internationalism in the social texture and international relations of the late Habsburg Empire. It appears that Von Suttner mobilized notions of the pacific effects of science with an eye to preserving both the European system of states and the position of the aristocracy.

## Introduction

Current endeavors to globalize the history of science stand in stark contrast to the way science *used to* be considered global when the field was first established. One of its founders, George Sarton, for example, also adopted a worldwide perspective but on entirely different grounds. Whereas historians today study knowledge as being affected (or even produced) by intercultural exchanges and travels through diverse locales, Sarton took his subject to be *supra*cultural and independent from context. Science transcended the specificities of place and culture; it belonged to humanity as a whole, as “the only truly solid bond between infinitely diverse people.”^1^ And precisely for that universal quality, Sarton added, science was the ideal guide to international cooperation and world peace.

This idealization – which is sometimes called scientific internationalism – has a history of its own that is worth studying, especially in contrast to histories of real, on-the-ground circulations of knowledge.^2^ The notion that science transcends national difference and can foster international cooperation became particularly pertinent in the decades around 1900 (the “first era of globalization”), gaining popularity through international manifestations of science and technology such as the universal expositions. This development is receiving increasing interest by historians of science as well as historians of international relations who have stressed its political dimensions.^3^ Mark Mazower has classified scientific internationalism among the Christian, socialist, and other internationalisms of its time; Glenda Sluga has shown how its varieties were mobilized in institutions like the League of Nations; and several others have scrutinized the uses of forms of scientific and technological internationalism in contexts from Britain to Brazil to Belgium.^4^

Hence there is an increasing appreciation of scientific internationalism as a political force – not just a notion that scientists entertained but an ideology that was actively promoted to political ends, such as the justification of imperial projects. Still it is my contention that we can only really grasp the precise politics of scientific internationalism if we analyze how exactly it was conceived – how actors *imagined* that science actually promotes peaceful cooperation, and how such understandings related to more widely held worldviews. There is no single answer to these questions. For Sarton, as we have seen, it was the universal validity of scientific knowledge that made science a unifier, implying that those nations that produced most of it were in the lead.^5^ For the sociologist Robert K. Merton, by contrast, it was the behavioral norm of universalism that demanded international cooperation, and this norm was most likely to be upheld in democratic societies.^6^ Views differed, and understanding the specific ways in which science was deemed to advance international cooperation may further help us uncover the particular politics of scientific-internationalist utterances. For their technoscientific imaginaries invariably implied geopolitical imaginaries: views of science harbored visions of international relations, world order, and arrangements of states. Hence a close study of how scientific internationalism was imagined and represented may tell us a lot about how science was publicly perceived as a force in the world. As studies of scientific displays have recently pointed out, the public understanding of science is not only about science but also about its political potential.^7^

In the following I pursue a close study of how scientific internationalism was imagined by analyzing the 1911 novel *Der Menschheit Hochgedanken* (translated as *When Thoughts Will Soar*) by the Austrian Baroness Bertha von Suttner.^8^ Both book and author are significant. The book told the story of a conference of scientists and engineers who, by the sheer brilliance of their thought, promoted peace and prevented war. The author was a world-famous pacifist, 1905 Nobel Peace Prize laureate, and one of the most prominent leaders of the international peace movement.^9^ Hence the novel can be analyzed as an imaginative rendering of scientific internationalism, contextualized in the international politics of its time.

The peace movement brought together a range of different groups and individuals, many of them middle- and upper middle class, but also a conspicuous segment of the aristocracy. It had a radical wing, with a significant number of women activists (most of them suffragettes) who aimed at peace through fundamental changes of social relations, “in the home, the village, and the nation.”^10^ The more male-dominated moderate wing limited itself to advocating arbitration, the legal form of conflict resolution.^11^ Von Suttner belonged largely to the latter camp. She was an active advocate of arbitration, but alongside this role also sought to advance peace through her literary work, which highlighted the futility of war and promoted pacifist sentiments.^12^ Her first and most famous novel in that vein, *Die Waffen Nieder!* (*Lay Down Your Arms!*), had become an international bestseller immediately after its publication in 1889.^13^ But it was her last novel, *Der Menschheit Hochgedanken*, that fully centered on science.^14^

There are two main reasons why this novel is well suited for the kind of analysis I propose. First and most obviously, the book is *about* science advancing peace and hence directly reflects how Von Suttner imagined this process and the world in which it took place. The story stages the scientific conference in a rich sociocultural setting of plenary meetings, sociable interactions, and geopolitical tensions, which Von Suttner colorfully described, portraying the kind of society in which scientific internationalism would make its mark – in her imagination.

But the book is also a helpful source because it is *not only* a work of the imagination. Von Suttner was a naturalistic author, who, following Émile Zola, aimed at presenting a crisp and honest view of the world as it was. To this end she based her writing largely on her own life experiences, even to the extent that she modeled many characters after real friends and acquaintances – *Der Menschheit Hochgedanken* was in effect a *roman-à-clef*.^15^ Moreover, the novel was in many ways an extrapolation of her autobiography. Von Suttner wrote it right after she had finished her *Memoiren* (1909), which had ended with reflections on “the immediate future,” and *Der Menschheit Hochgedanken* continued as “a Romance of the Immediate Future” – as stated in its subtitle.^16^ Its story was set just a few years ahead (“The twentieth century is still ‘in its teens’”), and it projected what Von Suttner hoped her activities and those of her fellow pacifists and scientists were going to accomplish.^17^ It hence reveals how she thought science could affect her world and what social change this process would (and would not) engender.

As analysts we can thus combine the novel with details from von Suttner’s biography to achieve a richly textured, politically contextualized understanding of scientific internationalism. Admittedly this analysis will be limited to the author’s views, especially since the book did not sell well and reached relatively few readers.^18^ But there are reasons to assume a somewhat larger significance. Reviewers ascribed the novel’s lack of success generally to the quality of the storytelling but lauded the brand of pacifism it presented.^19^ This is interesting because while science was clearly central to this pacifism, Von Suttner did not comprehensively spell out how it operated and left its precise workings in peace-making implicit. She did this not in order to hide anything but because she presumed her public would understand – and she seems to have been right about that. Even if her qualities as a novelist had become less appreciated by 1911, as the peace movement’s most experienced and arguably most successful propagandist she still knew how to get a pacifist message across and hence used an imagery that resonated with her prospective audience.

In the following I will start with laying out the fictional story line, then examine exactly how it portrayed science’s pacifist potential, and finally turn to its political context via the book’s connections to its author’s life. As we will see, the aspirations Von Suttner expressed were closely tied to late Habsburg society and her position among its high aristocracy. My analysis ends with a reflection on the aristocratic roots of scientific internationalism.

## The princess and the scientists

Von Suttner had written a dozen novels before *Der Menschheit Hochgedanken* and many of them featured protagonists called Martha, Hanna, Donna, Eva or another name similar to Bertha. The heroine of this book was Franka, surnamed Garlett, a smart and beautiful young princess. Franka’s mother had been abandoned by her aristocratic family when she married a bourgeois man, and she and this *Bildungsbürger* had given their daughter a thoroughly liberal education. By the time she reached maturity, however, both parents had passed away, and at the novel’s opening Franka is ready to find a job and work for a living. Through a fortunate accident, however, she is rediscovered by her grandfather who is more open-minded than the other relatives and who takes her into his castle, Sielenburg. There she lives among her conservative aunts and uncles who openly dismiss her free-spiritedness and modernity. But her grandfather adores her, and when he also dies she finds herself the sole heir to his fortune. Franka decides to use this wealth, and to dedicate her life to the advancement of two noble causes: women’s education and world peace. She chooses not to get married and becomes a writer and public speaker on these subjects.

Here the story skips a few years, and the remaining 296 pages are devoted to the conference. At some point in “191–” the American philanthropist John Toker (a character based on Andrew Carnegie) invites the now famous Franka to his annual “Rose-Week,” in Lucerne, Switzerland, a meeting of brilliant scientists, engineers, and intellectuals, whose sole task is to venture bold ideas and “High Thinking,” and hence advance world peace. Franka is quickly taken up in this circle, but also meets her Sielenburg family again, who have come to sit in the audience. The rest of the novel describes the various conference events (lectures, dinners, experimental demonstrations) and the spectators’ responses to them. Here Von Suttner draws stark contrasts between the young, modern, and forward-looking speakers and the audience of old-guard European nobility, whose women are obsessed with marriage and whose men are obsessed with war. While Toker and his scientists are advancing peace, the various dignitaries in the audience are stuck in cynicism and the belief in the wholesome effects of a good “steel bath.” In fact, war *is* about to break out in Europe, and Toker’s people work hard to prevent it, with the help of the modern-minded Prince Victor Adolph, son of a German monarch. The culmination of the novel, however, is not this political drama, but the story of Franka’s heart. She is courted by both Victor Adolph and the conference star, Chlodwig Helmer, and in the end chooses her fellow speaker.

## A meeting of minds

What is remarkable about the novel is not so much the plot as von Suttner’s portrayal of the conference – she depicted it almost literally as a meeting of minds. Gathered at the lakeside resort, the only task of Toker’s scientists and engineers was to venture “bold ideas” and “soaring thoughts,” which, by nothing but their very brilliance, would pacify the world and stave off war.^20^ The congress community was not just a model but an instrument for international politics. Ideas would actively counterbalance war, humanity’s highest thought would by itself lift the world out of its belligerence – hence the book’s title, *Der Menschheit Hochgedanken*, meaning literally: humanity’s high thought.

**Figure 1.**
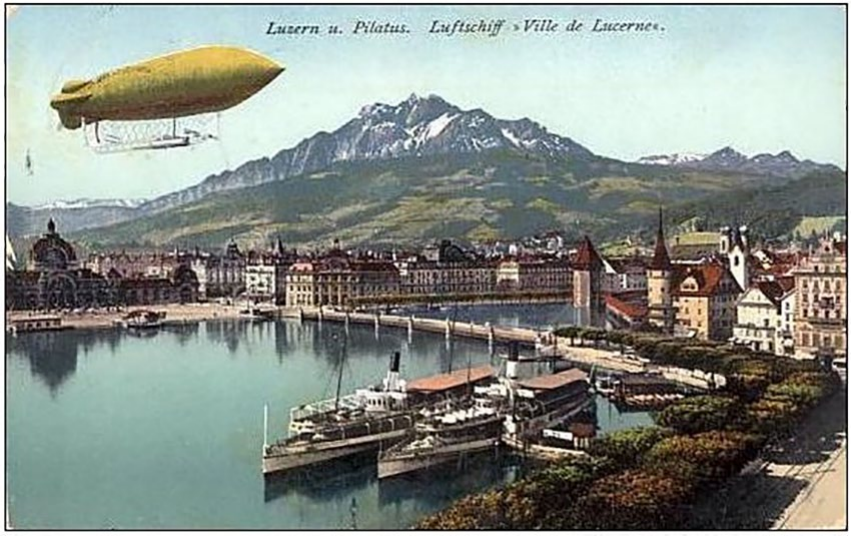
An actual dirigible over Lucerne, postcard from 1911.

**Figure 2.**
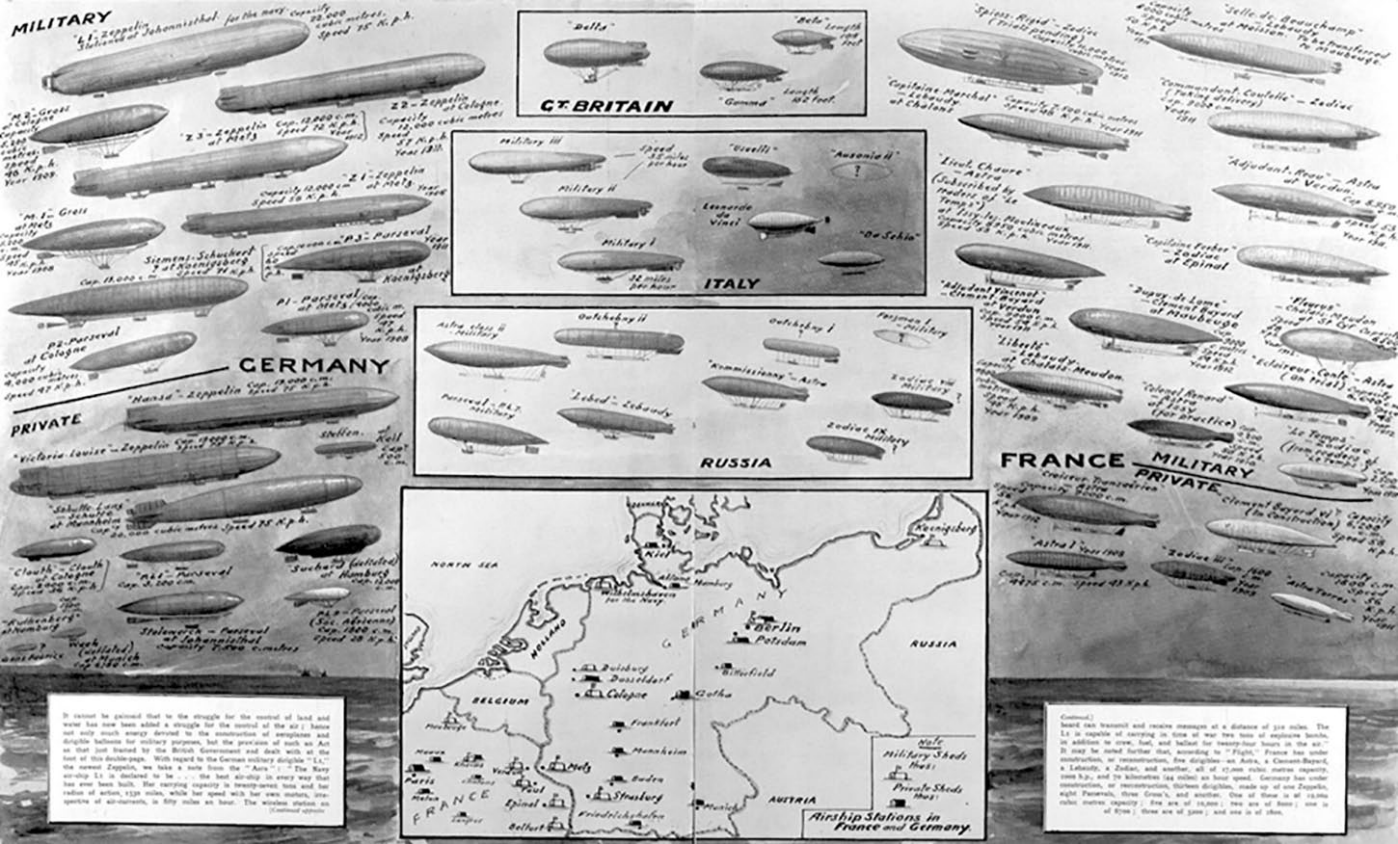
Great powers’ dirigible fleets chart, meant to induce fear in Britain. From *Illustrated London News* (22 February 1913).

Von Suttner related this belief in high thinking to a moral lag theory, the idea that technological progress has outpaced the development of morality and hence forms a potential menace to society.^21^ New technologies can be used for good as well as evil purposes and in order to make the right choices human ethics need to catch up with technological development. Von Suttner played this theory out in the form of a metaphorical comparison between physical and spiritual flight. She staged the conference against the backdrop of the introduction of dirigibles, the kind of propelled balloon airships that were very much in the public eye at the time of her writing (see Figures 1 and 2). Human flight was causing enormous excitement and its potential beneficial as well as destructive uses caught the popular imagination around 1910, for example in H. G. Wells’ prophetic *The War in the Air* (1908).^22^ Von Suttner knew Wells’ work well and it seems likely that this book inspired *Der Menschheit Hochgedanken* as an alternative future. Indeed it was the suggestion that airships could be turned into battleships that triggered the Toker character to organize the conference. His Rose-Week aimed to complement technological high flight with spiritual high thinking so as to catch up morally and attain the same altitude. Toker’s guests were supposed to do this mental high-flying and to produce the “thoughts that soar” from the English translation of the title.

The novel’s core activity, high thinking, was thus rather abstract, but Von Suttner made it happen in very concrete, and rather sumptuous, settings. Lodged in luxury hotels, the attendants met among lavish parks, with festal arches and grand lecture halls, all of which were copiously decorated with blooming roses, in garlands, escutcheons and flower beds – hence the conference’s name, Rose-Week. Pillars, tables, banquet halls, even the printed programs and invitations were adorned with the flowers, so that everywhere, the “intoxicating perfume of roses will fill all the air – a rose-bacchanal.”^23^ The participants were invited to simply stroll around and socialize with each other for the first week, after which, during the second week, they proclaimed their high thinking in a series of evening lectures and performances. These were accompanied by opera singing, theater, and at some point a sky dance of dirigibles, dressed up as Venetian gondolas, swans, and elegant sea ships, from which a young man and woman jumped out in newly invented wing-suits, diving and floating their way down toward the conference grounds, and then back up again into the airships.^24^ All second week performances took place before an audience of upper class tourists, who had flocked to the annual festival like they would attend a regatta or a horse race.^25^ At the same time the events and lectures were communicated to the larger public by newspaper journalists, through gramophone recordings, and by (silent) cinematographic reproductions, to be “sent out to thousands of schools and settlements all over the world.”^26^

These extravagant features make the Rose-Week look quite different than scientific conferences as we know them today. But, as Anne Rasmussen has pointed out, the conference was a rather novel phenomenon in the early twentieth century, and had not yet taken on the standard forms that we are familiar with. For one thing, a certain idealism was often associated with international scientific gatherings, which were often opened with extensive references to the pacifist nature of universal science.^27^ Also, the meaning of “congress” lay closer to that of parliament, and referred to the community of delegates rather than the meeting – one spoke of “sessions of the congress” in this or that discipline. Conferences were not just *tête-à-têtes* of specialist in-crowds, they carried messages to the public at large. These details do not explain all the characteristics of von Suttner’s lakeside gathering (which, for example, also included artists – about whom more will follow). But they do make sense of some of the features, such as the presence of an audience and the fact that in several passages Von Suttner called the collective of speakers the “Rose-Parliament.” She had never frequented scientific conferences herself but was familiar enough with the world of science to make such references.

## Thought for peace

Von Suttner extensively described the ways in which the soaring thoughts were expressed and communicated. But she did not spell out how the ideas would have real-world effects, how the mental high-flying would bring about international peace.^28^ At the same time she did pay ample attention to the thoughts expressed by the participants. The novel covers almost every lecture that speakers at the conference delivered, yet these exposés give few clues about the pacifying workings of mental power. One of her scientists, for example (a character resembling the biologist Ernst Haeckel as well as the chemist Wilhelm Ostwald), spoke about microorganisms, the beautiful world of the smallest creatures, and the physiology of aesthetic perception.^29^ An English engineer discussed the aerodynamics of the wing-suits that had just been demonstrated. Other lecturers talked about radium beams, soil fertilization, and new types of motor power – but never about their relation to peace. Nor did the artist speakers address international relations in any recognizable way. An Italian actress recited “Hero’s lament over the body of Leander.”^30^ Franka herself made a plea for men’s and women’s emancipation.^31^ And a “young Russian author” treated the audience to stories of suffering and persecution in his country in order to arouse feelings of pity, “that lofty feeling – one of the noblest of all.” For “indeed, I know the goal that beckons to the founder of this Rose-Congress. Lofty thoughts are to fly forth into the world; lofty feelings must be aroused.”^32^ Yet how loftiness led to peace remained unarticulated.

This point is underscored by the two longest lectures at the conference. Toker’s “speaker of the week” was Chlodwig Helmer, who offered reflections on the dilemmas of air flight.^33^ The new dirigibles were changing the game of international relations, and this held both wonderful and terrifying possibilities. The only way to ensure that good choices would be made was by raising our ethics equally high: “We are already able physically to soar up into the heights – we must do the same thing morally. We must learn to hold dominion over the realm of High Thinking.”^34^ But Helmer was silent about what this high thinking should entail. He merely repeated the moral lag theory that was the premise of Toker’s conference. The final speaker also presented new technological developments. With one hand outstretched to the audience, this young American engineer offered “gifts” to mankind in the form of news of great inventions that held the power to remake economic and social relations: fruits and vegetables of gigantic size to feed the poor, a “radium condenser” that could kill germs (or annihilate fleets), a marvelous diving suit, an air purifier, a hearing aid, and an arm strengthener.^35^ This speaker too stressed the ethical choices that these inventions raised, repeating the moral lag dilemma rather than showing a way out. Both he and Helmer restated the problem instead of solving it.

In the end, however, the content of the high thinking did not matter to Von Suttner. It was the height itself that produced peace. This is illustrated by her description of the very first lecture, a speech by a “great French author,” titled “La Verité, toute la Verité, rien que la Verité”:

Full of bold thought, of keen wit, of sparkling turns of speech, it was a bundle of new truths delivered to the auditors, and at the same time it was an unmasking of the lies that subjugate human society.^36^

Nothing else followed. This was all Von Suttner said about the lecture. She stressed the *kind* of thought that this great mind professed (bold, witty, true, and somehow critical of society) but gave no hints as to its contents. The brilliance of ideas counted, not their substance.

So then, how did brilliance lead to peace? How did Von Suttner imagine that great ideas and their spread among wider audiences would make a difference in international affairs? She let Toker ponder such issues at the end of the conference, where he himself concluded that thought would not be enough. Concrete measures were required, for “It is not sufficient that from here and there more ideas fly forth.”^37^ What followed was a plan of action:

[W]e must first train the whole race till it is fit for its new destiny. Practical work must be expanded in this direction. (. . .) Domestic colonization, garden-cities, hygiene along the whole line, extermination of the last vestige of illiteracy. And then, high schools will be established for the nurture of High Thinking and world-journals will be founded for its propaganda. (. . .) The problem must be worked out intensively, strenuously (. . .): as soon as I reach home, I intend to take measures to found the free academy of High Thinking.^38^

The inclusion of hygiene and garden cities in this list of measures may seem out of place, yet it was not unusual for turn-of-the-century pacifists to adopt this wider agenda of social reform. Pieter Eijkman, for example, a Dutch physician who proposed to build a World Capital around the Peace Palace in The Hague, was a hygienist, and designed that capital as a garden city.^39^ Hygienism and pacifism also went together in the American Progressive Movement, which produced among others Elihu Root, the arbitrationist and 1912 Nobel Peace laureate, who was also a character in the novel.^40^ Nevertheless, hygienic measures formed only a part of Toker’s plan. Most of it was about the dissemination of “High Thinking” through journals, schools, and academies – again begging the question of how this would produce peace.

Von Suttner did not eschew answering this question in order to build up suspense or for other literary reasons. The connection between high thought and world peace was self-evident to her, and, she suspected, self-evident to her readers. It is for this reason that she never explained that connection explicitly. Implicitly, however, her understanding of it can be gleaned from certain passages. Toward the end of the story, Toker and Victor Adolphe discussed the prospects of another peace conference in The Hague. Such conferences had taken place in the real world in 1899 and 1907.^41^ But even though they had been well-attended (almost all of the world’s states had sent high representatives), their results had been very meager, which left the modern German prince skeptical about another such event. But Toker was more optimistic, and his argumentation reveals a view of the causes of the prospects for peace. What had improved the chances since 1907 was, he argued, first of all, that war had become virtually unaffordable (partly because of the new need for air power). Second, public opinion was growing increasingly antibelligerent. But most importantly, since the last peace conference, an international spirit had been growing.

Since then, so many friendships, treaties, and conventions have arisen (. . .). Since then, all the groups interested have combined in an international organization. Since then, a world-conscience has come into being. Since then, the atmosphere has been conquered. Since then, human thoughts have attained wings. . . . Since then . . . “The old gentleman had worked himself into a fine heat; he had got up, and at every sentence his voice had grown louder. At the last ‘Since then,’ he suddenly stopped and sat down again. Then he went on in a calmer tone: – ‘Here we will pause – at the conception “Soaring Thoughts.”’ The delegates to the next conference are to be inspired with such thinking.^42^

In Toker’s view, the high thinking as it was practiced by his scientists and engineers was to inspire delegates to a new peace conference. Their soaring thoughts formed a “world-conscience” that would lift the spirits of those state representatives above national interests, enabling them “to accomplish something great, something bold” – such as the adoption of effective peace-making resolutions.^43^ Here lies a key to von Suttner’s understanding. For her, great ideas, irrespective of their content, had a supranational aspect. High thinking was by its nature elevated above the nation and forced those exposed to it to leave the national sphere. It was this geopolitical transcendence that could give spiritual abstractions real-world effects.

Von Suttner’s belief in the supranational nature of intellectual production reproduced the early modern ideal of the Republic of Letters, an ideal that had come to be rivaled by nationalist conceptions of science and culture during the nineteenth century, but was still available to her.^44^ In many ways, her community of conference speakers was itself a miniature Republic of Letters. How Von Suttner played out this conception, and why science and art were so central to her understanding of it, is illustrated by another passage in the novel. While the Rose-Week conference was proceeding, international tension had been building up, and by the end of it, a great war seemed imminent. In response, the “French senator” (based on von Suttner’s friend d’Estournelles de Constant) and the “American statesman” (the character resembling Elihu Root) proposed that the conference participants issue a joint statement, speaking out against the rising belligerence and urging those in power to prevent a conflict. But how could this be effective? Why would people give credence to scientists, engineers, and artists in matters of international affairs? The Frenchman explained:

Let us put aside false modesty; the Knighthood of the Rose must be conscious and ought to be conscious of its noble rank, in order to be forever mindful of the work to which it is pledged. John Toker summons only his contemporaries of world-wide reputation; only those who through their art, their scientific abilities, their inventions, their political activities, – particularly their service in the politics of peace, – have served all men, and therefore possess universal authority.^45^

The authority of the conference participants lay in their general brilliance but also, and especially, in their particular occupations. It was precisely *as* scientists, artists, and peace politicians that they could speak out with license. And the reason was that in these fields they “served all men.” Their work transcended private or national preoccupations by producing ideas that were gifts to humanity as a whole – much like the gifts in the American engineer’s speech. If the specific content of the high thinking was immaterial to Von Suttner, it did matter that it took place in science and in art, for only those fields (and pacifist politics) were geopolitically transcendent. Only scientists and artists possessed “universal authority,” and only “from their collected brilliancy a sudden enlightenment might gush out over the whole earth.”^46^

## Bertha/Franka

It is one thing to hermeneutically grasp von Suttner’s understanding of science and pacifism. It is another to situate these views in a wider social context and to relate them to the society she was presenting them to. Also for that purpose, the novel offers a wealth of clues as it was in many respects a projection of von Suttner’s own social environment. As said, she always aimed at naturalistic writing by staying close to her personal observations and experiences, and as a consequence her work deliberately reflected the world that she herself knew and lived in.^47^ This was true for the locations, the journeys, the sociable interactions, as well as the characters and classes of people. By combining the novel’s stories with details of von Suttner’s own life, therefore, we can see what world she was responding to and what real circumstances she thought the pacifying effects of science would change.

To dig out these dimensions it is key to realize that the princess in *Der Menschheit Hochgedanken* was in many respects a self-portrait of the author. Apart from the age difference of about 40 years, numerous parallels ran between Bertha and Franka. Like Bertha, Franka was of noble descent, but had fallen into poverty through no fault of her own (orphanage in the book, a gambling mother in real life). Like Bertha, Franka did not get married too soon but kept control of her own life, choosing to work in order to make ends meet. Like Bertha, Franka had wealthy, elderly suitors whom she turned away in disgust (Freiherr Ludwig Malhof in the book, onetime fiancé Gustav Heine in real life). And like Bertha, Franka eventually eloped with a man of lower rank against the family’s wishes. Von Suttner also portrayed Franka as widely adored and breathtakingly beautiful – and even that was not a complete flight of fancy as at a younger age Bertha too had had many admirers.^48^ But most of all, Von Suttner projected in Franka what she saw as her own attitude to life. She was an educated, modern, independent woman, working for a higher cause, and dedicating her life to spreading its message.

**Figure 3.**
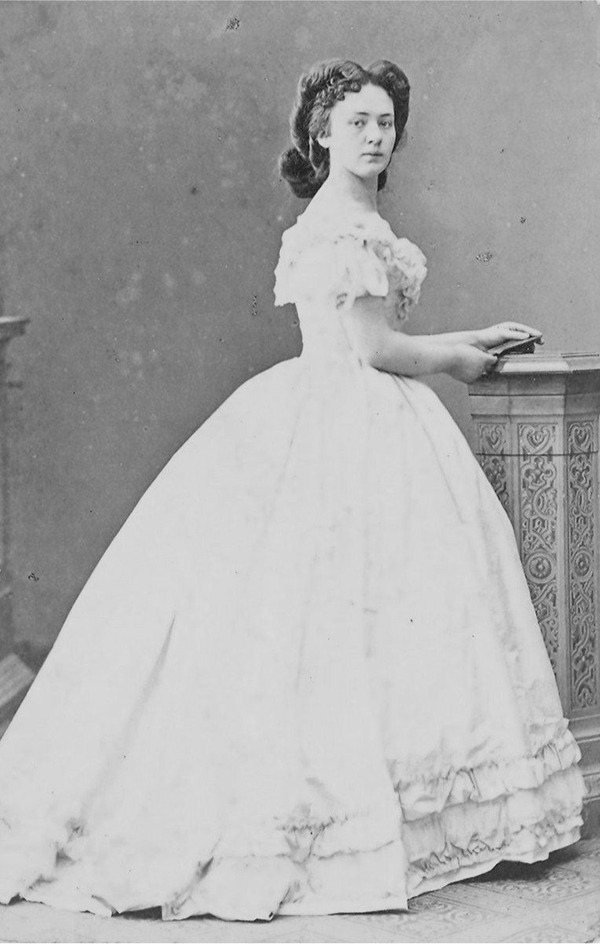
Bertha Kinsky at age thirty, 1873.

Importantly, however, Von Suttner had not always embraced this identity. In fact, she had spent the first thirty years of her life emulating a traditional aristocratic role model, aspiring to be a conventional Habsburg noblewoman (Figure 3). That had not been easy either. Her father had been an army general of the famous Bohemian House of Kinsky, who had died shortly before she was born. But her mother was fifty years younger and a commoner, which meant that even though Bertha could (and did) call herself “Gräfin Kinsky, von Wchinitz und Tettau,” she was in fact practically excluded from the higher aristocracy.^49^ Nevertheless most of her youth was dedicated to entering those circles anyhow, and Bertha was brought up in that expectation. Her mother and Aunt Loffe tried to bridge the financial gap at the casinos of Wiesbaden and Bad Homburg where they believed their clairvoyance could win them a fortune. When instead they lost most of their money, all hope was invested in marrying off Bertha to a “good match” of the upper classes. But poverty had estranged the family from society (they could simply not afford to go to many balls), and when Bertha finally made her *début* at a gala in Vienna, she was largely treated like an outsider. Several suitors did present themselves at other occasions, but luck was not on her side: one turned out to be a cheat, one died soon after their engagement (and was on the run from his creditors anyway), and one was seriously interested but so old that Bertha could not bring herself to bear his physical affections. By the age of thirty, she had become an “old maid,” destined to stay with her impoverished mother for the rest of her life.^50^

But there was one way in which Bertha’s excluded upbringing bore advantages: she was modernly educated and exceptionally well-read. Via her cousin Elvira, whose studious father had raised her on a diet of Hegel, Fichte, and Kant, Bertha had become interested in philosophy, science, and literature, and together the girls had read many books they would never have been exposed to in the convent schools that were customary for Austrian aristocratic daughters.^51^ Through governesses as well as the family’s travels, Bertha had also become fluent in French, Italian, and English, and she had taken music and singing lessons at fairly high levels. Altogether this was no professional education. But it did make her employable, and able to leave the dead end of spinsterdom by taking a job.

**Figure 4.**
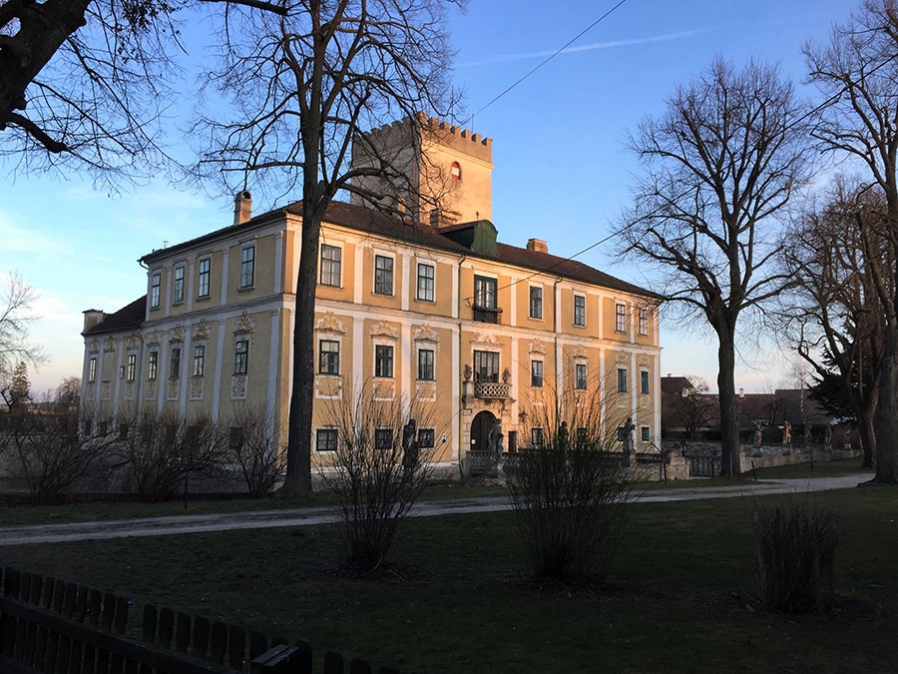
Schloß Harmannsdorf today.

It was here that new opportunities started to dawn upon Bertha and she began to carve out a new role – slowly and circuitously. In 1873, she became a governess with the wealthy industrialist von Suttner’s family in Vienna, and for three years she taught the four daughters – and fell in love with the son Arthur Gundaccar. This was a match quite different from Bertha’s previous ones, because Arthur was an “industrial baron” and not of the status she used to aspire to; at the same time, his parents would certainly forbid the marriage – Bertha’s own standing had sunk that far. When their secret engagement came out she was indeed forced to leave, and took a job in Paris. But before long, love drove her back to Vienna and the couple eloped to Georgia. There, they were received by Princess Ekatarine of the Dadiani royal family, whom Bertha had befriended a few years before. But with Arthur disowned, what followed were several difficult years in the Caucasus. He eked out a living working various odd jobs and Bertha tried to live off her novels. They both started to write articles on local subjects for various periodicals in Germany and Austria, and gradually acquired reputations as writers – she more than he. In the end Arthur’s parents reconciled themselves with their son’s marriage and in 1885 accepted him and Bertha back at their Schloß Harmannsdorf, which they eventually took over (Figure 4).

Bertha’s great breakthrough came a few years later. In 1887 she first heard of the international peace movement, and decided to contribute to its cause with a novel. This became *Die Waffen nieder!*, an instant commercial and critical success that was soon translated into a dozen languages. Almost overnight, Bertha became an internationally famous writer *cum* peace activist, whose voice and leadership were much sought after. She was regularly invited for speaking engagements, she cofounded Austrian and German branches of the pacifist movement, and she became the editor of the monthly magazine *Die Waffen nieder* (later *Die Friedens-Warte*).^52^ Neither a spinster nor the mere wife of a nobleman, Bertha had become an intellectual and political agent in her own right. She had become like Franka.

The Franka character was therefore not so much a reflection of her person as a whole as a version of what she (felt she) had become during the second half of her life – and what she wished she had been from the start. Looking back, Von Suttner no longer identified with her earlier person, that shallow, eager debutante “Bertha Kinsky [who] hovers before me like a figure in a picture book.”^53^ The ticket out of that existence had been her education in art, philosophy, and especially science. Science not only gave her a chance to make her own living, it also provided an understanding of the world and its development. Around the time of her transition, von Suttner’s favorite reads were Darwin, Haeckel, Spencer, and especially Henry Thomas Buckle’s *History of Civilisation*, which applied evolutionary theory to human society.^54^ She now saw evolutionary progress everywhere around her: in nature, in personal development, and in social relationships. How far had we advanced since the days of “our poor ancestors [who] lived in mud huts, with an incoherent intellect, undeveloped mind, knowing no greater joy than that of delivering a blow to an enemy’s skull with a club.”^55^ It was not so much natural selection that had produced this improvement but the ongoing process of civilization, especially among the educated. Violence was the result of ignorance, war was irrationality, and science showed the way out of these primitive predilections. These considerations set Von Suttner on the path toward pacifism, and it was in this way that she started to associate science both with peace and with her own journey from failing ballroom girl to famous public intellectual.

## Conservative scientism

The shift of von Suttner’s identity was accompanied by a shift in social circles. At Harmannsdorf, she found herself among Arthur’s family – new aristocrats but with all the traditional values of the old ruling class, and no interest in her intellectual and political activity.^56^ But away from home, she was taken up into the leading group of the international peace movement and its frequent gatherings. She became a regular invitee of its annual universal peace congresses as well as other occasions where “the whole company” (in her words) came together again – luncheons in Cannes, soirées on Lake Lucerne, winter sojourns with the Prince of Monte Carlo, etcetera.^57^ Von Suttner was also sometimes invited to political circles – to Emperor Franz Joseph, for example, and, most notably, to the 1899 Hague Peace Congress (a gathering of state representatives, not activists), where she was the only female attendant.^58^ But it was in and around the pacifist company that she found the type of people she identified with: “interesting and distinguished contemporaries,” “prominent personages from princely, scientific, diplomatic, and artistic circles.”^59^ These included political peace activists such as the journalist William Stead and the aforementioned Baron d’Estournelles, but especially pacifist intellectuals such as the economist Frédéric Passy, the astronomer Wilhelm Förster, writers like Leo Tolstoi and Björnstjerne Björnson, and “philosophers of nature” such as Ernst Haeckel.^60^ This was “the world’s intellectual aristocracy” that became her preferred estate.^61^

These two social worlds that Von Suttner knew from her own life (the traditional Habsburg nobility and the new internationalist intelligentsia) were also clearly projected – and starkly contrasted – in *Der Menschheit Hochgedanken*. On the one hand were the conference speakers, Toker’s Rose-Parliament, a small band of forward-looking, peace-seeking great minds. Von Suttner portrayed them as young, modern, future-oriented, and embracing scientific and technological progress. On the other hand was a cast of old-fashioned thinkers who were directly modeled upon the types of notables that had dominated her former life – and still dominated the Harmannsdorf household. These appeared in the novel as Franka’s Sielenburg family and as members of the conference audience. Von Suttner used their ignorant responses to the “High Thinking” of the speakers to highlight the latter’s brilliance and also to add a comical note, for among the old guard were some of the most amusing characters of the story. They included Franka’s arch-conservative cousin Coriolan (ever dismissive of anything intellectual), her aunts Adele and Albertine (always concerned with Franka’s wedding prospects), the dirty old man Baron Malhof (sleazily trying to please Franka), the slick Italian diplomat Marchese Romeo Rinotti (mainly interested in the conquest of women), Helmer’s dim old school friend Baron Franz Bruning (lazily enjoying the conference’s “cosmopolitan variety show”^62^), and retired colonel Baron Gaston de la Rochère (a French *revanchard* ironside). Like Statler and Waldorf at the Muppet Show, they commented on the conference, the women losing themselves in gossipy small talk, and the men in petty political discussions (so-called *Kannegiessereien*) about diplomatic intrigue and military preparation.^63^ War, for most of them, was the inevitable consequence of the natural struggle of states and could also have a wholesome effect on a society degenerating with modern ideas. As De la Rochère put it: “the old order and the sacred traditions are so shaken that only a good war could possibly set things straight again.”^64^

Von Suttner left little doubt as to the social station and political leanings of this class of people. They were Europe’s old aristocracy, conservative and militarist – “*vieux jeu*” and “*ancien régime*,” in her exact words.^65^ From a current historian’s vantage point, some of their nationalism and social Darwinism could seem quite up-to-date for 1911. But Von Suttner portrayed them as hopelessly lagging behind, belonging to a premodern era.

What is less obvious is where the politics of her high thinking heroes lay. They were clearly progressive and anti-establishment, but the nature and extent of their desire for change is not as clear-cut as it might appear. For one, von Suttner’s alter-ego Franka preached women’s education and emancipation but emphatically distanced herself from feminism. Just like the author eschewed association with suffragettes so did her protagonist – whose soul mate Helmer literally stressed: “Fräulein Garlett is no ‘*Feminist*’.”^66^ Similarly, the engineer who presented “gifts” that could feed the poor and benefit the lower social classes claimed that he was by no means a socialist: “do not be alarmed – I am not going to preach socialism.”^67^ What he aimed at was to elevate the poor from misery, but not by any movement from below. “The masses” and “the proletariat” appeared as a topic of conversation throughout the novel, but there were no indications that they would have anything more than a passive share in the making of the future.

In many conference conversations, Franka, Toker, and even Prince Victor Adolphe appeared as deeply concerned about poverty and “the social question,” and as seeing the poor as potential allies in their fight against war. But they also thought that the trouble with the masses was that they were ignorant and confused and hence without political direction. They would not act (and should not act) for lack of a coherent understanding of things. “I myself should not like to see the control of government put into the hands of the masses” declared Helmer, speaking to the Prince; first “[t]he general level of all mankind must rise.”^68^ The French senator agreed: “The masses, for the most part, wherever there is any thought at all, belong to the [pacifist camp], but they are dumb and as yet powerless.”^69^

The point was to lead the masses by inspiring them by with the right ideas – and this presented an opportunity for the high-thinkers of the Rose-Week. After a frustrating conversation with his old-fashioned school friend Bruning, Helmer felt discouraged by the dominance of conservatives – but then realized: “No, that is not true. We also have millions behind us – dumb, yearning millions, who are only waiting for the liberating act. The liberating act, however, must be forestalled by the liberating word . . . so let us first say just what we have to say.”^70^ In his opening speech, Toker similarly declared that the masses may be moved in the right direction by presenting them with proper and attractive ideas – the type of “soaring thought” that his conference would send out into the world.^71^ At the same time, change might be brought about top-down, by first enlightening “the rulers of human society.”^72^ As Helmer ‘poetically’ declared to the Prince: “I cherish the faith that by this time among the potentates, one – the Zeppelin – is born [who will pick up the lofty ideas] and will let his ship mount up into those heights of light.”^73^

All in all then, von Suttner’s modernists were very elitist. They were an avant-garde leading the masses, but certainly did not spring from them. If they were not literally identical to the old aristocracy, they did fit right into its place and share many of its characteristics. The comparison frequently appeared, throughout the novel. Toker called his conference participants “Rose-Knights,” the “Knighthood of the Rose,” and “the most noble assembly of kingly personages.”^74^ The French senator found them even more select than the aristocracy.^75^ In von Suttner’s direct description: “here were assembled the élite among men, who looked down from the higher pinnacles on the course of the world; who based their judgment on philosophical criteria and their will on humane sentiments.”^76^ Even the novel’s title, *Der Menschheit Hochgedanken*, could also have been translated as “humanity’s *noble* thoughts.”^77^

What she envisioned, therefore, was much less the removal of the upper class than its repopulation by a different kind of member: younger, more science-minded, less resigned, and more optimistic about the improvability of the world. But not altogether less exclusive. For all von Suttner’s emphasis on the youth and progressiveness of her heroes, they kept functioning within an old order that they wanted to repair, not replace. This observation raises questions about von Suttner’s alleged political convictions. According to her biographer, Brigitte Hamann, these were “firmly planted in the liberal political camp.”^78^ But as Daniel Laqua has recently pointed out, the Habsburg liberal camp was itself quite divided, and had a strained and ambiguous relation with pacifism.^79^ What Von Suttner did share with most liberal Austrians was a bourgeois ethos of education, modernity, and scientific progress. Deborah Coen has shown that Habsburg liberals generally cultivated scientific education in a rejection of the Catholic dogmatism of the right and the politics of class and national division of the left.^80^ This was also true for Von Suttner: her internationalism was directed against nationalist separatism (more about this follows) while her reading of Haeckel and Buckle was definitely anti-Catholic. But her scientific outlook did not imply a dismissal of the *other* pillars of the right, the monarchy and the aristocracy – let alone a restructuring of the social order as a whole. In her novels, she defended the position of the emperor, and while she criticized the Austrian upper class for being closed off to new blood, she at the same time praised the English nobility for its openness to reform itself.^81^

**Figure 5.**
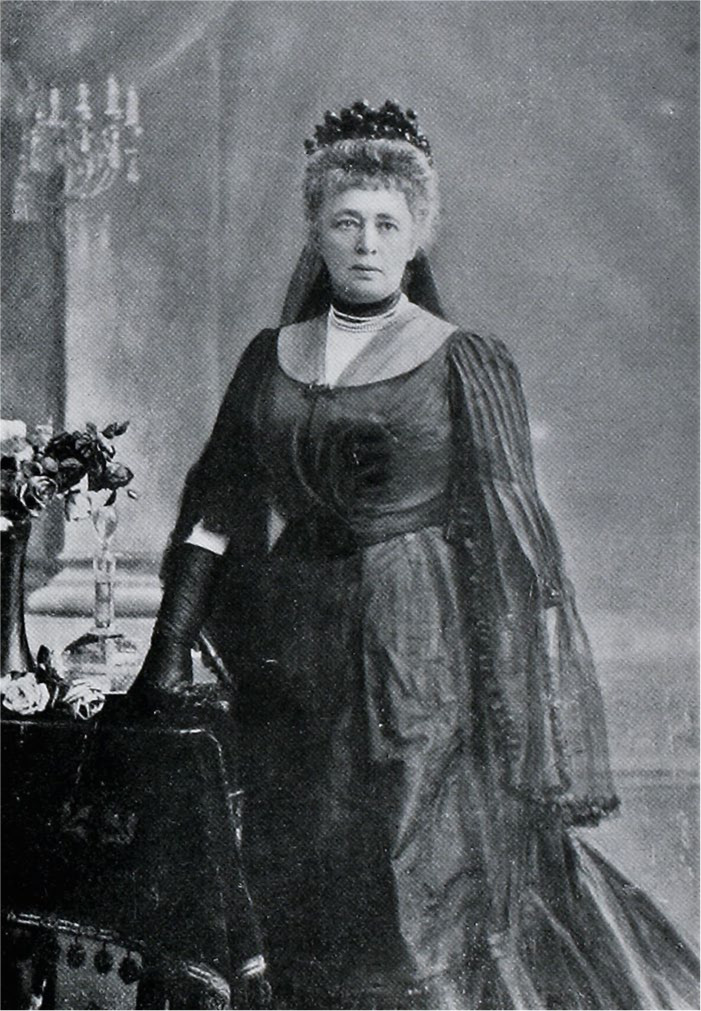
Von Suttner in 1913, with crown and in mourning dress.

All in all, Von Suttner still much esteemed the value of *an* aristocracy – one that was composed of a reformed, yet still elite, type of people. Unlike in, for example, H. G. Wells’ writings such an avant-garde was not produced by selective breeding (Von Suttner was no eugenicist) but by progressive civilization.^82^ The main protagonists in *Die Waffen nieder!* underwent a reformation from “nobility” (*Edelleute*) to “noble people” (*Edelmenschen*).^83^ But while in this process their enlightenment increased, their social position stayed the same, their class did not vanish. Von Suttner never lost her appreciation for the upper class, ever since her youth as an aspiring debutante. The luxury and refinement, the good taste and great wealth, the leadership that naturally came with position – these were never things she despised or wanted to get rid of (Figure 5). A scientific mindset was needed to modernize the ruling class in order to *preserve* its legitimacy as a social stratum. Science was not pitched against but meant to sustain the hierarchies of the Habsburg *ancien régime*.

## Habsburg universalism

Besides the vertical social order of class divisions, *Der Menschheit Hochgedanken* also commented on the horizontal social order of international relations, the system of states. This is amusingly suggested by a passage toward the end of the story. The conference was set circa 1918 or 1919, but suddenly, on one of its final evenings, incognito in the audience, appeared Archduke Franz Ferdinand, the crown prince and pretender to the Habsburg throne.^84^ He was alive and well. In von Suttner’s imagination, no shot would ever be fired in Sarajevo, no world war was to take place. Of course she could not know that such a conflict would come about (as it happened she would die exactly one week before the archduke did). But it is important to realize that an upheaval like it was expected in *Der Menschheit Hochgedanken*, and the novel’s scientists and engineers did everything to prevent it. Through their efforts, they effectively preserved the Habsburg Empire, the Hohenzollern Empire, and the Romanov, Ottoman, British, and French empires – in short, the entire old order as it existed. It was this world order that scientific “High Thinking” was meant to sustain.

Von Suttner’s science-driven pacifism was not geared toward transforming the state system of Europe, it was meant to preserve the status quo. An aspect of this attitude became apparent in her reporting of the 1892 Universal Peace Congress in Bern, Switzerland, where a Polish participant claimed that the organization should also strive for the restoration of Poland, a state violently snatched from its people by land-hungry belligerents. The leading pacifists disagreed. They answered that such an intervention could never be the aim of the movement, whose business it was to end border disputes, not to start them. Von Suttner recalled how Frédéric Passy especially had “laid down the law to the Polish patriot”:

[T]he Congress could not possibly occupy itself with the revision of Polish history. The justice of the future is to be made ready for; the individual injustices of history cannot now be rectified, for all the divisions of the land as at present constituted are based on the ground of force; new laws, new ordinances – and they must be worked for – have no retroactive power.^85^

Current states were not to be divided – Von Suttner fully endorsed this policy and dismissed international revisionism in other instances as well. In *Der Menschheit Hochgedanken*, some of the conference participants were of national minorities in Europe’s states, but none of their work for peace included claims for autonomy or independence. Von Suttner was no champion of self-determination, if that concept had any meaning to her at all. In her eyes, nationalism was not a liberating force, but one of the greatest threats to peace. She associated it not only with national separatist movements that were its greatest champions in Central Europe, but also with the arch-conservativism of De la Rochère, the old warhorse in the novel, who regarded patriotism as “the highest concept.”^86^

Colonial independence was something that barely even came up in Von Suttner’s writings. What was despicable about imperialism, according to her, was not the subjugation of populations – that was necessary to help them on the path of progress and civilization. It was the fact that imperial powers made war *on each other*. As civilized nations they should know better.^87^ Just as the “barbaric” practice of dueling for the sake of settling disputes between individuals had been replaced by reasonable settlement in a court of law, so resorting to military violence in international conflicts should be supplanted by rational resolution in a court of arbitration.^88^ That was the direction in which civilization and progress were pointing.

But if the creation of new borders was anathema to von Suttner’s pacifism, so was the erasure of existing ones. That war was to be abolished between the powers did not imply that they all should merge into a global unity. Current countries were there to stay, neither fragmenting into smaller national units (in the case of multinational empires) nor blurring into a single world state (as internationalists such as H. G. Wells advocated).^89^ Von Suttner believed in a middle ground, and that middle ground was exemplified by the supranational state that was the Habsburg Empire, a state that kept its external borders, yet whose internal diversity did not lead to internal division – at least in its ideal conception. In fact, nationalist agitation was dangerously challenging the empire’s integrity by the early twentieth century, and Von Suttner deeply deplored this. She saw herself as superseding such nationalism, and, in a sense, as predating it. At a speaking engagement in the Deutsches Haus in Prague, in 1895, she recited two (translated) Czech poets, was taken aback by the hostile reactions of the German audience, and subsequently rejoiced when it was won over by the sheer beauty of the poetry. Art had vanquished nationalist antagonism, and Habsburg supranational unity had been restored – in her interpretation of the event.^90^ Seven years later, Von Suttner spent a few summer weeks in the Bohemian countryside, when the “village people” surprised her with an homage. But the local school teacher delivered an address that she could neither understand nor reply to since she did not know the Czech language – even though she herself was of Bohemian descent. She realized,

I do not know my native tongue. To be sure the Kinskys are a Czechish family, but in my childhood the Czechish national consciousness had not [yet] awakened, and as I grew older I was no longer receptive to it, having attained the European consciousness.^91^

As Rita Krueger and others have observed, many Bohemian aristocrats had embraced a national identity by this time that was more or less antagonistic to their association with Vienna.^92^ But this was not the case for Von Suttner. Czech nationalism had risen *after* her upbringing in Bohemia, and during the wandering existence that followed she had not absorbed it anymore. But while the “European consciousness” she had thus acquired precluded such nationalization, it had not made her drop her Habsburg identity.

Von Suttner certainly regarded herself as internationally oriented, but that orientation matched with – and was an extrapolation of – the supranational nature of the Dual Monarchy. Deborah Coen has recently shown how Habsburg environmental scientists used the empire as a model for international organizations and conceptions.^93^ Furthermore Daniel Laqua has discussed the different responses of Habsburg pacifists to the rise of nationalisms within the empire. One option was to take the peaceful methods for resolving international conflict as a model for dealing with these internal disputes, which, in a sense, were no less inter-national.^94^ Von Suttner seems to have taken this position, except that she did not start from internationalism and “apply” it to the intraimperial situation, but rather the other way around: she took her identification with the supranational nature of the Habsburg Empire (and its ruling elite) and extended it to her internationalist and pacifist outlook. In her autobiography, she spoke admiringly of her stepfather, “Fritzerl” Fürstenberg, “a type of the old-fashioned Austrian,” whose worldview also predated national divisions and in many ways transcended them.^95^ The supranational state that he represented was the reasonable middle ground between nationalist fragmentation and cosmopolitan dissolution – the ideal building block of the world order that von Suttner’s science-based pacifism sought to preserve.

## Aristocratic internationalism

Historians have long associated the belle-époque peace movement with bourgeois liberalism.^96^ There can indeed be little doubt that its beliefs in civilization, progress, and scientific rationality derived largely from that camp. However, many of the movement’s members were in fact aristocrats, sometimes even royalty, and hence it would be surprising not to also find some of this stratum’s values and attitudes seeping through in its pacifism and internationalism. Karina Urbach recently made this point by identifying Europe’s aristocrats as internationalists *par excellence*. More than any other class, they were equipped to act as informal go-betweens for diplomatic purposes, for three main reasons. Most of the nobility were used to speaking several languages, and did so fluently. Many of them were unusually well-traveled, and had family ties that crossed many borders (as did their employability in military and state services). And all of them shared a social code of honor, courtesy, and devotion to ancestral cults. According to Urbach, “Before the term *Weltbürger* (citizen of the world) was invented, the ‘aristocrat of the world’ existed.”^97^

Many of these qualities can readily be recognized in Von Suttner. She spoke German, French, Italian, and English from very early on. As a child, she spent summers in Venice, Paris, Switzerland, and other places (a fact that she pointed out – not without a tinge of pride – as contrasting with her originally bourgeois husband who only started traveling after they were married).^98^ And she shared the particular comportment, the ways of “holding oneself,” that came with an aristocratic upbringing and that lubricated her foreign relations, also with fellow (often aristocrat) pacifists. For Von Suttner, precisely these traits made this class of people into “the ennobled type” of a new nation that would conquer the world, “the nation of cosmopolitans.”^99^

Von Suttner’s internationalism was not something that she only acquired after she became interested in science, modern ideas, and the peace movement, and then carried over to her way of life as a noblewoman. It was, importantly, something she had already been brought up into, had lived and carried with her, precisely as an aristocrat. As a consequence we should not see her pacifism as stemming from her scientific outlook, and being used to sustain the old order and the upper class. It was also very much a product of that class. The internationalism that Von Suttner associated with science was not inherent in it, but brought to it, by her, from a different background. Hence her life and work suggest that, perhaps more than we would tend to think, the scientific internationalism of her time was heir to the aristocracy.

## Conclusion

“Few political notions are at once so normative and so equivocal as internationalism,” Perry Anderson observed at the start of the millennium. “It is claimed on all sides as a value, but who can identify it without challenge?”^100^ His words seem to hold *a forteriori* for scientific variety: commonly praised as beneficial, but rarely defined in concrete terms. Now, much has changed since Anderson made his observation. Several historians have pinpointed the specific political programs that lay behind the generic term. But this is less true for scientific internationalism. Many have identified it as a form of internationalism but then set it aside *next to* various political types, hence obscuring the particular politics that it might hold.

In this article I have tried to trace the particular politics of scientific internationalism in one important actor’s expressions. As I have argued, the best way to do this is to scrutinize how precisely science was *imagined* to have pacifying effects. In von Suttner’s novel it was the communal character of scientific discoveries – the notion that they were “gifts to humanity” – that made science unifying. Moreover, the novel showed what this *meant* in the author’s world. Von Suttner portrayed the bringers of these gifts – scientists – as a new noble elite. Her vision of a desirable future was one led by an aristocracy – not the old upper class that she knew and that had rejected her, but a new aristocracy of scientific-internationalist minds, like the one that had adopted her into the peace movement. Her aim was this replacement – but within a social structure that was still very much like the Habsburg *ancien régime*. In a comparable way, Von Suttner wanted to reform international relations – from antagonistic and rooted in military values to cooperative and based on peaceful understanding. But these changes too should not upend the existing system of states, but happen within it, and thus sustain it, following the multinational Habsburg model.

A novel is no philosophical treatise or political party program – certainly this novel was not, with its family melodrama and tacky love stories. But it can still be read for its political imaginaries, for two main reasons. First, Von Suttner did write the book with political intentions. Like all of her novels they were meant to inspire pacifist attitudes, in this case by ridiculing those who believed in war as dimwitted and out of date and by portraying pacifists as young and attractive, forward-looking, and science-minded. But Von Suttner’s politics can also be found in the unintentional, in the conceptions she did not explain (or did not even consider explaining) because they seemed self-evident to her. This is true for her view of world order, the system of states that pacifism was to sustain. It also applies to her idea of how science could pacify the world, and, at least to an extent, to her vision of the aristocracy as a necessary, exemplary, leading class. Reading works of fiction can hence help to grasp an author’s mental universe, and, in the case of a politically influential author like Bertha von Suttner, help to understand an important strand of politics and the role of science within it. Novels are key to technoscientific and geopolitical imaginaries.

## Notes

1. George Sarton, “L’Histoire de la science,” *Isis* 1 (1913): 3–46, 43 (my translation).

2. This sense of the term should be distinguished from another usage: scientific internationalism as a body of practices in international science, such as conferences, organizational work, etc. The latter usage was more common at the time, e.g., in the catalogue of such activities, *L’Internationalisme scientifique* (The Hague: Stichting voor Internationalisme, 1911) by P. H. Eijkman. In this article I stick to the former meaning.

3. Robert Fox, *Science without Frontiers: Cosmopolitanism and National Interests in the World of Learning, 1870–1940* (Corvallis: Oregon State University Press, 2016); Harmke Kamminga and Geert Somsen (eds.), *Pursuing the Unity of Science: Ideology and Scientific Practice from the Great War to the Cold War* (Aldershot: Ashgate Publishing, 2016); W. Boyd Rayward (ed.), *Information Beyond Borders: International Cultural and Intellectual Exchange in the Belle Époque* (Aldershot: Ashgate Publishing, 2014); Fabian de Kloe, *Constructing Worlds with Words: Science and International Language in the Early Twentieth Century* (PhD diss., Maastricht University, 2014); Rebecka Lettevall, Sven Widmalm and Geert Somsen (eds.), *Neutrality in Twentieth-Century Europe: Intersections of Science, Culture and Politics after the First World War* (New York and London: Routledge, 2012); Jessica Reinisch and David Brydan (eds.), *Europe’s Internationalists: Rethinking the History of Internationalism* (London and New York: Bloomsbury Academic, 2021).

4. Mark Mazower, *Governing the World. The History of an Idea, 1815 to the Present* (New York: Penguin Press, 2012); Glenda Sluga, *Internationalism in an Age of Nationalism* (Philadelphia: University of Pennsylvania Press, 2013); Johan Schot and Vincent Lagendijk, “Technocratic Internationalism in the Interwar Years: Building Europe on Motorways and Electricity Networks,” *Journal of Modern European History* 6 (2008): 196–217; Thomas Mougey, “Tracing the Origins of Brazil’s Great Acceleration: The SPVEA’s Primeiro Plano Quinquenal and the Technoscientific Recovery of Amazonia, 1945–1959,” *Varia Historia* 34 (2018): 375–408; Thomas Mougey, “Needham at the Crossroads: History, Politics and International Science in Wartime China (1942–1946),” *British Journal for the History of Science* 50 (2017): 83–109; Waqar Zaidi, “‘Aviation Will Either Destroy or Save Our Civilization’: Proposals for the International Control of Aviation, 1920–45,” *Journal of Contemporary History* 46 (2011): 150–78; Waqar Zaidi, “‘A Blessing in Disguise’: Reconstructing International Relations Through Atomic Energy, 1945–1948,” *Past and Present* 210 (2011): 309–31; Daniel Laqua, *The Age of Internationalism and Belgium, 1880–1930: Peace, Progress and Prestige* (Manchester: Manchester University Press, 2013); Geert Somsen, “Scientists of the World Unite: Socialist Internationalism and the Unity of Science,” in Kamminga and Somsen (eds.), *Pursuing the Unity of Science*, pp.82–108 (note 3).

5. This is how Sarton thought the torch of (universal) civilization was passed on. See e.g., his *The Incubation of Western Culture in the Middle East* (Washington, DC: Library of Congress, 1951).

6. Robert K. Merton, “Science and Technology in a Democratic Order,” *Journal of Legal and Political Sociology* 1 (1942): 115–26; Cf. David A. Hollinger, “The Defense of Democracy and Robert K. Merton’s Formulation of the Scientific Ethos,” *Knowledge and Society* 4 (1983): 1–15.

7. Jaume Sastre-Juan, “Philanthropy, Mass Media and Cultural Hegemony: the Rockefeller Foundation and the Politics of Science Popularization in the 1930s,” in Massimiliano Badino and Pietro Daniel Omodeo (eds.), *Gramsci Today: Cultural Hegemony in a Scientific World* (Leiden: Brill, forthcoming); Elena Canadelli, Marco Beretta and Laura Ronzon (eds.), *Behind the Exhibit: Displaying Science and Technology at World’s Fairs and Museums in the Twentieth Century* (Washington, DC: Smithsonian Institution Scholarly Press, 2019); Robert Kargon, Karen Fiss, Morris Low and Arthur Molella (eds.), *World’s Fairs on the Eve of War. Science, Technology, and Modernity, 1937–1942* (Pittsburgh: University of Pittsburgh Press, 2015).

8. Bertha von Suttner, *Der Menschheit Hochgedanken: Roman aus der nächsten Zukunft* (Berlin: Verlag der “Friedens-warte,” 1911), translated as *When Thoughts Will Soar: a Romance of the Immediate Future*, trans. into English by Nathan Haskell Dole (Boston and New York: Houghton Mifflin Company, 1914). I will quote from this English translation.

9. There is vast literature on Von Suttner, and a string of biographies, many hagiographic. The best biography to date is Brigitte Hamann, *Bertha von Suttner. A Life for Peace*, trans. into English by Ann Dubsky (Syracuse: Syracuse University Press, 1996).

10. Sandi E. Cooper, “French Feminists and Pacifism, 1889–1914: The Evolution of New Visions,” *Peace & Change* 36 (2011): 5–33, 8; *Patriotic Pacifism: Waging War on War in Europe, 1815–1914* (New York: Oxford University Press, 1991).

11. See e.g., Sandi E. Cooper, “The Impact of Nationalism on European Peace Movements, 1848*–*1914,” *Peace & Change* 6 (1980): 23–6. Some of the moderates saw arbitration itself as scientific but this is not how Von Suttner portrayed the pacific effects of science. Daniel Laqua, “Alfred H. Fried and the Challenges for ‘Scientific Pacifism’ in the Belle Époque,” in Rayward, *Information Beyond Borders*, pp.181–99 (note 3). Geert Somsen, “Global Government through Science: Pieter Eijkman’s Plans for a World Capital,” in Rayward, *Information Beyond Borders*, pp.201–19 (note 3).

12. Bertha von Suttner, *Memoirs. Records of an Eventful Life*, trans. into English by authorized translation (Boston and London: Ginn and Company, 1910), Vol. 1:294–5.

13. Bertha von Suttner, *Die Waffen nieder! Eine Lebensgeschichte* (Dresden: E. Pierson’s Verlag, 1889); *Lay Down Your Arms*, trans. into English by T. Holmes (London: Longmans & Co., 1892).

14. So far the novel has received scant scholarly attention. Exceptions are Beatrix Kempf, *Suffragette for Peace. The Life of Bertha von Suttner* trans. into English by R.W. Last (London: Ostwald Wolff, 1972), pp.77–81; Anne Stalfort, “*Das Maschinenzeitalter* und *Der Menschheit Hochgedanken*. Bertha von Suttners literarische Utopien” in Ina Breuckel et al. (eds.), *Bei Gefahr des Untergangs: Phantasien des Aufbrechens; Festschrift für Irmgard Roebling* (Würzburg: Königshausen & Neumann, 2000), pp.197–217; and Regina Braker, “Bertha von Suttner as Author. The Harriet Beecher Stowe of the Peace Movement,” *Peace & Change* 16 (1991): 74–96.

15. Hamann, *Bertha von Suttner*, pp.40, 51–2 (note 9). The novel’s heroes especially were based on people she knew and admired, such as Thomas Edison and George Bernard Shaw. I identify others in the following sections.

16. Bertha von Suttner, *Memoiren* (Stuttgart und Leipzig: Deutsche Verlags-Anstalt, 1909), p.542.

17. Von Suttner, *When Thoughts Will Soar*, p.139 (note 8). These aims are comparable to those of H. G. Wells in *Anticipations* (New York and London: Harper & brothers, 1902) and *A Modern Utopia* (London: Nelson and sons, 1905), namely to produce imagined but “sincerely intended forecast[s] of the way things will probably go” (*Anticipations*, p.3).

18. In fact Von Suttner had had great trouble selling her manuscript and found only a friendly pacifist publisher to be interested. See Kempf, *Suffragette for Peace*, pp.79–80 (note 14).

19. See reviews of the book and its English translation (anonymous unless specified) in: *The American Review of Reviews* 50 (1914): 248; *The Advocate of Peace* 76 (1914): 267; Elsbeth Friedrichs-Jena, *Die Friedens-Warte* 13 (1911): 355–7; and *The New York Times*, 21 June 1914.

20. Von Suttner, *When Thoughts Will Soar*, pp.212, 156 (note 8).

21. Moral lag theories were ubiquitous in international relations theorizing in general. See e.g., Waqar Zaidi, *Technology and the Reconstruction of International Relations: Liberal Internationalist Proposals for the Internationalisation of Aviation and the International Control of Atomic Energy in Britain, USA and France, 1920–1950* (PhD diss., Imperial College, University of London, 2008), ch. 1.

22. H. G. Wells, *The War in the Air* (London: G. Bell, 1908). On the cultural impact of flight and its moral ambivalences, see Robert Wohl, *A Passion for Wings: Aviation and the Western Imagination, 1908–1918* (New Haven: Yale University Press, 1994); David Edgerton, *England and the Aeroplane: An Essay on a Militant and Technological Nation* (London: Macmillan, 1991); and Bernhard Rieger, *Technology and the Culture of Modernity in Britain and Germany, 1890–1945* (Cambridge: Cambridge University Press, 2005).

23. Von Suttner, *When Thoughts Will Soar*, p.67 (note 8).

24. Ibid., pp.272–3.

25. Ibid., p.65.

26. Ibid., p.66.

27. Anne Rasmussen, “Jalons pour une histoire des Congrès Internationaux au XIX^e^ siècle: Régulation scientifique et propagande intellectuelle,” *Relations Internationales* 62 (1990): 115–33, 124–6.

28. In fact Von Suttner had one of the (critical) audience members raise this very question: “how can the world be made better by a few self-conceited people making speeches before a few other frivolous people? The only effect that addresses have on me is to make me sleepy.” Von Suttner, *When Thoughts Will Soar*, p.151 (note 8).

29. Ibid., pp.358–9. The depiction of the beauty of microorganisms is highly reminiscent of Haeckel’s *Kunstformen der Natur*, 2 Vols. (Leipzig and Vienna: Verlag des Bibliographisches Instituts, 1904). But the real Haeckel also appears in the novel as a subject of conversation. For the identification with Ostwald, see Thomas Hapke, “Wilhelm Ostwald, the ‘Brücke’ (Bridge), and Connections to Other Bibliographic Activities at the Beginning of the Twentieth Century,” in Mary Ellen Bowden, Trudi Bellardo Hahn and Robert V. Williams (eds.), *Proceedings of the 1998 Conference on the History and Heritage of Science Information Systems* (Medford: Information Today, 1999), pp.139–47, 144. By the time Von Suttner was writing, she knew Ostwald as a scholar of wide competence, a popular writer, and an active internationalist – all characteristics that fit her novel’s character.

30. Von Suttner, *When Thoughts Will Soar*, p.291 (note 8).

31. Ibid., pp.246–53.

32. Ibid., p.232.

33. Ibid., p.295.

34. Ibid., pp.305–6.

35. Ibid., pp.411–13.

36. Ibid., p.226.

37. Ibid., p.437.

38. Ibid.

39. Somsen, “Global Government,” pp.201–19 (note 11).

40. See Von Suttner, *When Thoughts Will Soar*, p.174 (note 8). Richard Bartholdt, the president of the Interparliamentary Union, the greatest international arbitration advocacy group, was another prominent peace activist from the Progressive Movement. On hygienism generally, see Roy Porter, *The Greatest Benefit to Mankind. A Medical History of Humanity from Antiquity to the Present* (Fulham: Fontana Press, 1999), pp.397–427.

41. See e.g., Arthur Eyffinger and Pieter Hendrik Kooijmans, *The 1899 Hague Peace Conference: the Parliament of Man, the Federation of the World* (The Hague: Kluwer Law International, 1999) and Arthur Eyffinger, *The 1907 Hague Peace Conference: the Conscience of the Civilized World* (The Hague: Judicap, 2007).

42. Von Suttner, *When Thoughts Will Soar*, p.375 (note 8).

43. Ibid.

44. Geert J. Somsen, “A History of Universalism: Conceptions of the Internationality of Science, 1750–1950,” *Minerva* 46 (2008): 361–79, 363–7.

45. Von Suttner, *When Thoughts Will Soar*, p.386 (note 8).

46. Ibid., p.154. These were Toker’s words.

47. See Hamann, *von Suttner*, pp.40, 51–2 (note 9).

48. For biographical details, see Hamann, *von Suttner* (note 9).

49. Hamann, *von Suttner*, pp.2–3 (note 9). Most of the following details are based on this biography.

50. Ibid., pp.5–17.

51. Ibid., p.18. Von Suttner, *Memoirs*, 1:20 (note 12).

52. Besides Hamann, *von Suttner* (note 9), see Kempf, *Suffragette for Peace* (note 14); Christian Göltz, *Die Rebellin. Botschaften fur unsere Zeit* (Elsdorf: Klein und Blechinger, 1996), pp.88–115 esp.; and Marie-Claire Hoock-Demarle, *Bertha von Suttner 1843–1914: Amazone de la paix* (Villeneuve d’Asq: Presses Universitaires du Septentrion, 2014), esp. chs. 6, 7, 9, and 10.

53. Von Suttner, *Memoirs*, 1:192 (note 12).

54. Ibid., 1: 249–50. On Buckle’s influence on von Suttner’s writing, see Helen G. Morris-Keitel, “(R)Evolution: From *Edelleute* to *Edelmenschen* – Bertha von Suttner’s Pathway to Peace,” *Seminar. A Journal of Germanic Studies* 50 (2014): 34–50.

55. Quoted in Hamann, *von Suttner*, p.40 (note 9).

56. Ibid., pp.48–9.

57. Bertha von Suttner, *Memoirs. Records of an Eventful Life*, trans. into English by authorized translation (Boston and London: Ginn and Company, 1910), Vol. 2:392, 401.

58. Ibid., pp.157–8, 245–326. Von Suttner published a personal report of her experiences at The Hague as *Die Haager Friedenskonferenz. Tagebuchblätter von Bertha von Suttner* (Dresden and Leipzig: E. Pierson’s Verlag, 1900).

59. Von Suttner, *Memoirs*, 1:3 (note 12); Von Suttner, *Memoirs*, 2:401 (note 57).

60. The quote is from Von Suttner, *Memoirs*, 1:248 (note 12).

61. Ibid., 1:xii.

62. Von Suttner, *When Thoughts Will Soar*, p.193 (note 8).

63. Ibid., p.384.

64. Ibid., pp.178–9.

65. In the words of the coquettish young widow Annette Felsen, addressing De la Rochère. Ibid., pp.178–9 (original italics).

66. Ibid., p.183 (original italics). On von Suttner’s feminism, see e.g., Laurie R. Cohen, “‘Fighting for Peace amid Paralyzed Popular Opinion’: Bertha von Suttner’s and Rosa Mayreder’s Pacifist-Feminst Insights on Gender, War and Peace,” in Bruna Bianchi and Geraldine Ludbrook (eds.), *Living War, Thinking Peace (1914–1924): Women’s Experiences, Feminist Thought, and International Relations* (Newcastle upon Tyne: Cambridge Scholars Publishing, 2016), pp.108–22.

67. Von Suttner, *When Thoughts Will Soar*, p.403 (note 8).

68. Ibid., p.337.

69. Ibid., p.385.

70. Ibid., p.196.

71. Ibid., pp.155–6.

72. Ibid., p.169.

73. Ibid., p.339.

74. Ibid., p.145.

75. Ibid., p.386.

76. Ibid., p.384.

77. I thank one anonymous reviewer for this suggestion. It could be that the American translator deliberately eschewed the aristocratic reference but there are no explicit indications of this.

78. Hamann, *von Suttner*, p.38 (note 9). Some of the older literature tends to read a progressivism back into belle époque actors like Von Suttner. Regina Braker even suggests that “[i]t is not clear that she did not sympathize with socialist ideas.” Braker, “von Suttner,” p.84 (note 14).

79. Daniel Laqua, “Pacifism in Fin-de-Siècle Austria: The Politics and Limits of Peace Activism,” *The Historical Journal* 57 (2014): 199–224.

80. Deborah Coen, *Vienna in the Age of Uncertainty. Science, Liberalism, and Private Life* (Chicago and London: The University of Chicago Press, 2007), pp.10–15.

81. Morris-Keitel, “(R)Evolution,” pp.46–7 (note 54). Hamann, *von Suttner*, p.3 (note 9).

82. See e.g., Wells, *Anticipations* (note 17).

83. Morris-Keitel, “(R)Evolution” (note 54).

84. Von Suttner, *When Thoughts Will Soar*, pp.289–90 (note 8).

85. Von Suttner, *Memoirs*, 1:431 (note 12).

86. Von Suttner, *When Thoughts Will Soar*, p.178 (note 8).

87. For comparable views by fellow internationalists (and Nobel Peace laureates), see Adam Tooze, *The Deluge. The Great War and the Remaking of Global Order, 1916–1931* (London: Penguin Books, 2015), pp.60–1 and Stephen Wertheim, “Reluctant Liberator: Theodore Roosevelt’s Philosophy of Self-Government and Preparation for Philippine Independence,” *Presidential Studies Quarterly* 39 (2009): 494–518.

88. The comparison of war with dueling, and the notion of their abolishment with the advancement of civilization, was common among pacifists of the time. Andrew Carnegie, on whom the John Toker character was based, elaborated this in writings such as *A League of Peace* (New York: The New York Peace Society, 1911). Antidueling leagues were also formed in pacifist circles. See Von Suttner, *Memoirs*, 1:137–8 (note 12); and Von Suttner, *Memoirs*, 2:392–4 (note 57).

89. Among his many expressions of this ideal, see e.g., H. G. Wells, *A Modern Utopia* (London: Nelson and sons, 1905).

90. Von Suttner, *Memoirs*, 2:82 (note 57).

91. Ibid., 2:395–6. Von Suttner in the end did answer the school teacher via a translator.

92. Rita Krueger, *Czech, German, and Noble Status and National Identity in Habsburg Bohemia* (New York: Oxford University Press, 2009); Eagle Glassheim, *Noble Nationalists. The Transformation of the Bohemian Aristocracy* (Cambridge, MA and London: Harvard University Press, 2005).

93. Deborah R. Coen, *Climate in Motion. Science, Empire, and the Problem of Scale* (Chicago and London: The University of Chicago Press, 2018), pp.356–8.

94. Laqua, “Pacifism in Fin-de-Siècle Austria,” pp.217–21 (note 79). Laqua associates Von Suttner with another position (the choice of abstinence), but also shows that she supported Czech–German reconciliation. Her associate Alfred Fried even maintained that the Germans had laid “the cultural foundations of this empire.” Cited on p.220.

95. Von Suttner, *Memoirs*, 1:11 (note 12).

96. The classic statement is from Roger Chickering, *Imperial Germany and a World Without War. The Peace Movement and German Society, 1892–1914* (Princeton: Princeton University Press, 1975), pp.25–6, who called late nineteenth-century pacifism “an extreme political variety of liberal (as opposed to revolutionary) internationalism,” and put it at the center of a Venn diagram of liberal internationalisms. See also Fredrik Egefur, “Who Should Fight for Peace? The Bourgeois Peace Movement and the Women’s Movement Before the First World War,” in Anders Ahlbäck (ed.), *Gender, War and Peace: Breaking Up the Boundaries* (Joensuu: University Press of Eastern Finland, 2014), pp.60–73; and Hamann, *von Suttner*, p.38 (note 9).

97. Karina Urbach, *Go-Betweens for Hitler* (New York: Oxford University Press, 2015), p.9. Dina Gusejnova has argued that aristocrats were even instrumental in carrying over supranational perspectives to early European integration projects, *European Elites and Ideas of Empire, 1917–1957* (Cambridge: Cambridge University Press, 2016).

98. In her memoirs, Von Suttner stressed how new Switzerland, Paris, and many other places already familiar to her were to Arthur Gundaccar. E.g., on Asia: “A different continent – it fills the comparatively inexperienced traveler with a peculiar pride, a pride upon which old globe-trotters look down with a smile. My Own [von Suttner’s pet name for her husband] set his foot on the un-European soil with the haughtiness of a conqueror.” Von Suttner, *Memoirs*, 1:218 (note 12).

99. From Bertha von Suttner, *High Life* (München: O. Heinrichs, 1886), quoted in Gerhard Danzer, *Europa, deine Frauen. Beiträge zu einer weiblichen Kulturgeschichte* (Berlin and Heidelberg: Springer Verlag, 2015), pp.249–50, my translation. Danzer reads this passage as a plea for education, but it is in fact an education in aristocratic conduct.

100. Perry Anderson, “Internationalism: A Breviary,” *New Left Review* 14 (2002): 5–25, 5.

